# Dengue virus infection: potential applications of "Omics" based approaches

**DOI:** 10.6026/973206300200802

**Published:** 2024-07-31

**Authors:** Hind A. Alkhatabi, Alaa G. Alahmadi, Reem M. Alotibi, Mohammad H. Alhashmi, Ammar A. Basabrain, Peter Natesan Pushparaj

**Affiliations:** 1 Department of Biochemistry, College of Science, University of Jeddah, Jeddah, 21959, Saudi Arabia; 2 Center of Excellence in Genomic Medicine Research, King Abdulaziz University, Jeddah 21589, Saudi Arabia; 3 Department of Medical Laboratory Technology, Faculty of Applied Medical Sciences, King Abdulaziz University, Jeddah 21589, Saudi Arabia; 4 Toxicology and Forensic Sciences Unit, King Fahd Medical Research Center, King Abdulaziz University, Jeddah 22254, Saudi Arabia; 5 Hematology Research Unit, King Fahd Medical Research Center, King Abdulaziz University, Jeddah 21589, Saudi Arabia

**Keywords:** Dengue fever, hemorrhage, shock, flavivirus, cytokine storm, biomarkers, Omics

## Abstract

Dengue, caused by the dengue virus (DENV), a member of the flavivirus family, continues to pose a significant threat to populations worldwide, despite advances in technology. Nearly half of the global population is at risk of contracting the disease, ranging from mild dengue fever (DF) to severe dengue hemorrhagic fever (DHF) and Dengue Shock Syndrome (DSS). The precise mechanisms underlying the progression of DF to DHF and DSS remain unclear and the presence of various DENV serotypes exacerbates this situation. Urbanization and climate change are expected to affect dengue epidemiology, potentially increasing the frequency and intensity of outbreaks. This review aims to consolidate the current knowledge on the biological characteristics, pathogenesis and application of "Omics" based strategies for biomarker discovery for precision medicine. Although the precise mechanisms behind the progression from DF to DHF/DSS are not fully understood, hypotheses include immune over-activation, cytokine storms and antibody-dependent enhancement. Studies of comorbid conditions have shown no significant association with the development of DHF/DSS in patients with diabetes, hypertension, or other chronic diseases. Despite the far-reaching and intricate nature of dengue, the inconsistencies found in clinical pathophysiological studies underscore the need for additional research aimed at elucidating the pathogenesis of DHF/DSS and devising effective preventive measures. Identifying the differentially expressed genes, proteins and metabolites in DF, DHF and DSS may enrich our understanding of the mechanisms underlying their pathogenesis. Moreover, these differentially regulated pathways may serve as novel therapeutic targets. These biomarkers may also be utilized for disease surveillance and the evaluation of the efficacy of therapeutic interventions for personalized treatment. Continuous research is essential to gain deeper insights into the mechanisms and progression of dengue fever and to formulate more effective prevention and control strategies. A multidisciplinary approach is vital for comprehending dengue virus pathogenesis, identifying risk factors and creating targeted interventions, particularly through biomarker discovery using "Omics" approaches.

## Background:

Dengue virus (DENV) is a rapidly emerging mosquito-borne viral disease that poses a significant threat to global public health [1]. According to the World Health Organization (WHO), more than 100 countries are endemic for DENV transmission and approximately 3.5 billion people living in tropical and subtropical regions are at risk of contracting the virus [2]. On average, there are approximately 50 million DENV infections and 500,000 hospitalizations annually [2]. The mortality rate for DENV infections is approximately 5%, with children and young adults being particularly susceptible [3]. DENV is a positive-sense, single-stranded RNA virus that belongs to the genus Flavivirus and family Flaviviridae. It comprises four distinct serotypes (DENV 1, 2, 3 and 4). Although these serotypes are serologically related, they are antigenically distinct from each other. The envelope proteins of DENV serotypes share common epitopes, making it difficult to identify specific strains of the virus using serological tests [4]. DENV has a diameter of approximately 40-60 nm and is spherical with a nucleocapsid core measuring 30 nm in diameter [1]. The nucleocapsid is enclosed by a envelope that is 10 nm thick. The nucleocapsid contains core proteins and viral genomic RNA [5]. The genome is a non-segmented, single-stranded, positive-sense RNA 10.7 kb in length and shares approximately 70% of its sequence with the four virus serotypes. Consequently, different genotypes are clustered, encoding three structural proteins (C, capsid; prM/M, precursor of membrane/membrane; E, envelope) and seven non-structural proteins (NS1, NS2A, NS2B, NS3, NS4A, NS4B and NS5) [6].

## Dengue virus infection and virus cycle:

Female mosquitoes belonging to the genus Aedes are responsible for transmitting the virus from human hosts to other individuals during the febrile period, which occurs prior to the completion of the feeding process (Figure 1A - see PDF). DENV infection can result in a range of clinical illnesses, including classical dengue fever (DF), dengue hemorrhagic fever (DHF) and dengue shock syndrome (DSS), which can vary in severity and may be life-threatening [2]. DF typically manifests with a sudden onset of fever, headache, retro-orbital pain and myalgia, while DHF and DSS are characterized by hemorrhagic symptoms, plasma leakage and severe shock (Figure 1B - see PDF). Antibody-dependent enhancement (ADE) of DENV replication significantly contributes to the severity of DHF and DSS [5]. A. aegypti distribution without dengue epidemic and DF deaths in the year 2012 per million individuals are depicted in Figure 1C (see PDF) and ID (see PDF), respectively. Upon maturation, dengue viral particles attach to host cells and gain entry via a process known as endocytosis. Subsequently, the viral and endosomal membranes fuse and release the viral genome. The genome is then translated into proteins that initiate the replication process. The proteins produced by the dengue virus (DENV) interact with cellular lipids and can be secreted. The virus matures in the endoplasmic reticulum (ER) and is subsequently released from the host cell. It is important to note that some of the particles released during this process are immature and non-infectious [7] (Figure 2 - see PDF).

Infection with one DENV serotype does not confer protection against the other serotypes. Moreover, subsequent infections can significantly increase the risk of developing DHF and DSS, and all four DENV serotypes possess antigenic combinations rather than distinct serotypes [4] (Figure 3 - see PDF).

## Host immunity and molecular mechanism of infection:

The development of DHF and DSS is primarily attributed to the potency of the virus and irregular host immune responses [8]. Upon entering a human host through the bite of an infected mosquito, DENV infects via the mannose receptor on macrophages and dendritic cell-specific ICAM-3-grabbing non-integrin 1 (DC-SIGN) on dendritic cells [5]. This action triggers the activation of innate immune cells, including mast cells, which initiate a local inflammatory response to the virus in the skin, attracting leukocytes such as natural killer (NK) cells and cytotoxic T cells, to eliminate virus-infected cells at the site of injection. However, infected dendritic cells mature and migrate to local or regional lymph nodes where they present viral antigens to T cells. The infected cells release viruses that infect and replicate inside monocytes/macrophages, B cells, other dendritic cells and Langerhans cells [9]. (Figure 4 - see PDF) DENV replicates inside the immune cells, leading to viremia [1]. The virus enters the bloodstream, likely via infected B cells, in the draining lymph nodes and infects other organs, such as the liver, kidneys and spleen. In contrast, DENV infection activates memory T lymphocytes (CD4+ T Cells) and induces the release of interferon gamma (IFN-g), which further up regulates Fc Receptors (FcR) on monocytes and propagates DENV infection [5]. The secretion of vasoactive mediators by infected immune cells causes vascular permeability, which may lead to DSS (Figure 4 - see PDF). An array of cytokines and other mediators, such as platelet activation factor (PAF), complement activation components (C3a and C5a) and histamine, increase hemorrhage and vascular permeability. DENV infection causes infected cells to display viral antigens in conjunction with major histocompatibility complex (MHC) antigens, leading to the activation and stimulation of CD4+ and CD8+ T cells. Tumor necrosis factor alpha (TNFα) increases vascular permeability and individuals with the TNFα308A allele, which is associated with increased production of this cytokine, are more susceptible to developing DHF [5]. Autoimmune reactions have been suggested to play a role in the development of DHF and DSS from DF [10]. The DENV envelope protein contains a 20-amino acid sequence that shares similar amino acid residues with a group of clotting factors, as demonstrated by the presence of cross-reactive antibodies to plasminogen in DENV infections. These antibodies have been associated with haemorrhagic manifestations. Research has shown that higher levels of platelet activation IgG (PAIgG) are closely related to thrombocytopenia during the acute phase of secondary infection [10].

Bibliometric analysis of dengue and omics based strategies A bibliometric analysis utilizing the Scopus database was conducted to determine the quantity of documents, including articles, reviews and book chapters, pertaining to "Dengue" and "Omics" over the past 20 years (Figure 5 - see PDF). The results revealed a surprisingly low number of 37 documents related to both subjects. Furthermore, no studies were identified that employed a combination of Genomics, Proteomics and Metabolomics to discover molecular markers for predicting disease progression from DF to DHF and DSS. This indicates a significant gap in knowledge in this area. However, recent studies, such as the analysis of serum metabolites in DF and multiplex cytokine analysis have been conducted [11, 12]. To address this knowledge gap, we propose the use of cutting-edge high-throughput technologies, such as Luminex xMAP technology by Luminex Corporation (USA) and Liquid Chromatography combined with Synapt G2 Mass Spectrometry by Waters Corporation (USA), in conjunction with data analysis using Functional Bioinformatics Strategies. By identifying novel molecular markers, healthcare professionals will be able to develop more effective treatment strategies for DF, DHF and DSS, which can significantly improve patient outcomes, reduce hospitalization rates and decrease healthcare expenditures as demonstrated previously [13, 14]. DF is a complex ailment for clinical diagnosis owing to its resemblance to other febrile diseases, including influenza, chikungunya, malaria and leptospirosis, Hantavirus and Salmonella typhimurium [10]. The main diagnostic tools for DF are serum biomarkers such as viral components and antibodies, including Immunoglobulin M (IgM) and Immunoglobulin G (IgG). In DHF and DSS, the antibody response is classified based on the severity of the disease, such as high IgM titer with low anti-flavivirus IgG titer, low IgM titer with high anti-flavivirus IgG titer and high IgM titer with high anti-flavivirus IgG titer [10]. However, relying solely on clinical diagnosis is insufficient for predicting the progression of DF to DHF and DSS. It is crucial to recognize that infection with one DENV serotype results in lifelong immunity but does not guarantee protection against other serotypes of DENV [15].

## Conclusion and Future directions:

Dengue infection has a significant effect on societies and economies in many countries, making it a critical focus for controlling emerging infectious diseases. Unfortunately, epidemiological studies on this topic are scarce. The integration of data from Genomics, Proteomics and Metabolomics approaches could lead to the identification of novel molecular markers that could be used to design personalized treatments for DF, DHF and DSS. By employing a high-throughput genomics, proteomics and metabolomics approach in conjunction with functional bioinformatics, it is possible to identify and validate druggable molecular markers for different severities of Dengue virus infection. This comprehensive approach not only enhances our understanding of dengue pathogenesis but also paves the way for personalized treatment measures tailored to individual patients with DF, DHF and DSS.
